# Temozolomide as salvage treatment in primary brain lymphomas

**DOI:** 10.1038/sj.bjc.6603660

**Published:** 2007-02-27

**Authors:** M Reni, F Zaja, W Mason, J Perry, E Mazza, M Spina, R Bordonaro, F Ilariucci, M Faedi, G Corazzelli, P Manno, E Franceschi, A Pace, M Candela, A Abbadessa, C Stelitano, G Latte, A J M Ferreri

**Affiliations:** 1 Medical Oncology Unit, Department of Oncology, San Raffaele Scientific Institute, Milan, Italy; 2 Department of Hematology, Clinica Ematologica, Policlinico Universitario, Udine, Italy; 3 Department of Medicine, Princess Margaret Hospital, Toronto, Ontario, Canada; 4 Department of Medicine, Bayview Regional Cancer Center, Toronto, Ontario, Canada; 5 Department of Medical Oncology, Division of Medical Oncology National Cancer Institute, Aviano, Italy; 6 Department of Oncology, S Luigi Currò Hospital, Catania, Italy; 7 Department of Oncology, Spallanzani Hospital, Reggio Emilia, Italy; 8 Department of Oncology, M Bufalini G Marconi Hospital, Cesena, Italy; 9 Department of Hematology, Unità Operativa Ematologia Oncologica, Istituto Nazionale Tumori, Fondazione ‘G Pascale’ IRCCS, Neaples, Italy; 10 Department of Oncology, Oncologia Medica, Ospedale Civile Maggiore-Borgo Trento, Verona, Italy; 11 Department of Medical Oncology, Bellaria Hospital Bologna, Italy; 12 Department of Neuroscience, ‘Regina Elena’ Institute, Rome, Italy; 13 Department of Medicine, Clinica Medica, Torrette Hospital, Ancona, Italy; 14 Department of Internal Medicine–Naples, Onco-Hematology Unit, S Sebastiano Hospital and Division of Internal Medicine, Second University of Neaples, Italy; 15 Department of Hematology–Azienda Ospedaliera Bianchi-Melacrino-Morelli, Division of Hematology, Reggio Calabria, Italy; 16 Department of Hematology, Division of Hematology, S Francesco Hospital, Nuoro, Italy

**Keywords:** phase II trial, primary brain lymphoma, salvage therapy, temozolomide

## Abstract

Methotrexate (MTX)-based chemotherapy extends survival in patients with primary brain lymphomas, but it is not clear whether multiagent chemotherapy is superior to MTX alone. Treatment options for patients with recurrent primary brain lymphoma are limited; there is no standard second-line chemotherapy. New chemotherapeutic agents with clear activity in brain lymphoma are needed for treatment of recurrent disease. We report the results of a phase II trial assessing activity of the alkylating agent temozolomide in immunocompetent patients with recurrent primary brain lymphomas, previously treated with high-dose MTX-containing chemotherapy and/or radiotherapy. A median of two courses (range 1–12) of temozolomide 150 mg m^−2^ day^−1^, for 5 days every 4 weeks was administered to 36 patients yielding nine complete and two partial responses (response rate: 31%; 95% confidence interval 16–46%). One-year survival was 31% (95% confidence interval 16–46%). Toxicity was negligible. We conclude that temozolomide is active in recurrent primary brain lymphomas and should further be evaluated in this disease, perhaps in combination with MTX as initial treatment.

There is general consensus that high-dose methotrexate (HD-MTX) is the cornerstone of the initial treatment of primary central nervous system lymphomas (PCNSL) (Reni *et al*, 1997; Ferreri *et al*, 2000, 2002, 2003; Reni and Ferreri, 2004a). However, for most patients, this remains an incurable disease, and attempts to improve survival with combinations of HD-MTX and other chemotherapeutic agents have not convincingly been shown to be superior to MTX alone (Ferreri *et al*, 2002; Ferreri *et al*, 2003). Although a recent retrospective analysis has suggested that the addition of cytarabine to HD-MTX might be an independent positive prognostic factor for improved survival (Ferreri *et al*, 2002), this observation has not been confirmed in a randomised phase III trial, in part because of the difficulties associated with conducting large prospective studies in such a rare disease. There are few agents with demonstrable activity in primary brain lymphoma. Most active agents used for extracerebral non-Hodgkin's lymphomas do not penetrate the blood–brain barrier at sufficient concentration to be effective against primary brain lymphoma, and agents that do penetrate the CNS have generally not been very effective, or have caused unacceptable toxicities (Ferreri *et al*, 2003; Reni and Ferreri, 2004a). As salvage therapy improves survival in PCNSL (Reni *et al*, 1999), we have chosen to prospectively evaluate new drugs in patients with relapsed or refractory disease in an attempt to identify promising agents with activity in primary brain lymphoma. This paper reports the final results of a phase II trial assessing the activity of a single-agent temozolomide for patients with recurrent PCNSL. Preliminary results of this trial and our rationale for choosing temozolomide for evaluation in PCNSL have been reported elsewhere (Reni *et al*, 2004b).

## MATERIALS AND METHODS

Patients were eligible for enrolment in this trial provided they met the following criteria: age >17 years, failure following initial treatment with HD-MTX and or radiotherapy, histologic diagnosis of PCNSL, presence of at least one bi-dimensionally measurable target lesion, negative HIV serology, ECOG performance status (PS) <4, adequate bone marrow (platelet⩾100 000 mm^3^, haemoglobin ⩾10 g dl^−1^, absolute neutrophil count ⩾1500 mm^3^), renal (serum creatinine ⩾2 times upper limit of normal (UNL)) and hepatic function (SGOT/SGPT ⩾3 times UNL, bilirubin and alkaline phosphatase ⩾2 times UNL). The protocol was reviewed and approved by local ethics committees. All participating patients provided written informed consent. The study was conducted in agreement with the Declaration of Helsinki. Temozolomide was administered at 150 mg m^−2^ day^−1^, for 5 days every 4 weeks until progression of disease (PD), unacceptable toxicity or patient's refusal. Temozolomide was administered for a maximum of six cycles when the best response was stabilisation of the disease (SD). In patients with objective response, at least two cycles of temozolomide were administered after maximum radiographic response. Criteria for dose modification in the event of toxicity have been described previously (Reni *et al*, 2004b). Briefly, for absolute neutrophil count ⩾1500 mm^−3^ or platelets ⩾100 000 mm^−3^ on the intended day of re-treatment, the start of the next cycle was delayed until haematopoietic recovery for a maximum of 2 weeks. For grade 3 or 4 toxicity, dosage for subsequent cycles of temozolomide was reduced to 100 mg m^−2^.

Pretreatment evaluation included whole body computed tomography (CT) scan, whole brain CT (*N*=6) or magnetic resonance (MR; *N*=30) scan, and, whenever possible, CSF examination with cell count and cytology. Whole brain CT or MR scans were repeated every 2 months during chemotherapy and every 3 months thereafter. Toxicity was graded by the National Cancer Institute Common Toxicity Criteria version 2.0 and response to treatment was assessed according to the criteria of MacDonald *et al* (1990). The best response recorded from the start of the treatment was considered. The progression-free and overall survival were measured from initiation of treatment. All analyses were performed on an intent-to-treat basis. The principal end point of this trial was the objective radiographic response rate to temozolomide. The maximum response rate considered of low interest was 15% and the minimum response rate considered of interest was 35%. The target enrolment (*α*=0.05; *β*=0.10) was estimated to be 38 patients, among whom at least 10 objective responses were necessary to declare temozolomide active against PCNSL.

## RESULTS

After enrolment of 36 patients, nine complete responses (25; 95% confidence interval 11–39%) and two partial responses (6; 95% confidence interval 0–14%) were observed. The study was closed at this time as the target of 10 objective responses was achieved. Patient characteristics (January 2000–June 2005) and previous treatment are summarised in Table 1. Twenty-eight patients (78%) had a first recurrence of PCNSL and eight had multiple recurrent disease having failed other salvage regimens. Median age was 60 years (range 34–81) and median ECOG performance status was 2 years (range 0–4). Median complete response duration was 7 months (range 1–70+) and four patients are currently free of disease at 7, 17.5, 20 and 70 months. All responses but one were observed among patients who were monitored by MR imaging. One complete response was observed among six patients monitored by CT scan and this lasted 6 months. Five patients had SD, 14 had PD and six died before response could be evaluated, probably due to PD. The clinical course of the five patients with SD was variable, likely owing to further therapy whose details were not available after PD in two patients (nos. 8 and 21) and to lymphoplasmacytic histology in one patient (no. 8). Since completion of our trial, new response criteria for PCNSL have been suggested (Abrey *et al*, 2005). No change in response rates was observed when these criteria were applied to our series. Five patients were alive at a median follow-up of 22 months (range 14–74) after initial failure, two patients were lost to follow-up with PD at 22.5 and 25.5 months and 29 patients had died. Median progression-free survival was 2.8 months (interquartile range 1–8 months), median overall survival was 3.9 months (interquartile range 1.7–16 months) and 1-year overall survival was 31% (95% confidence interval 16–46%). Altogether, 125 cycles (median 2; range 1–12) of temozolomide were delivered. Toxicity was mild; two patients had one episode of grade 4 neutropenia, associated in one case with grade 4 thrombocytopenia, and one patient had grade 3 vomiting in a single cycle.

## DISCUSSION

The final results of this trial of temozolomide monotherapy for recurrent PCNSL confirm and extend our preliminary observations (Reni and Ferreri, 2004a). As this is the first phase II trial of salvage monochemotherapy for PCNSL, we arbitrarily chose a minimum response rate of 35% for a drug to be considered of interest. We observed an objective response rate of 31%, most of which were complete responses, in a heavily pretreated patient population, with poor PS. These results suggest that temozolomide is an active agent against PCNSL. Recent reports of outcomes for recurrent PCNSL using combined chemotherapy (Arellano-Rodrigo *et al*, 2003; Tyson *et al*, 2003), single agent topotecan (Fischer *et al*, 2006) or combined chemo-immunotherapy (Enting *et al*, 2004) have observed responses similar to those we observed with temozolomide. In these studies, reported response rates were 33–53% and 1-year survivals were 25–58% (Arellano-Rodrigo *et al*, 2003; Tyson *et al*, 2003; Enting *et al*, 2004; Fischer *et al*, 2006) with better results observed in the smallest retrospective series, which included almost exclusively (93%) recurrent patients (Enting *et al*, 2004). The conclusions one can draw from these series are limited because in most cases less than 20 patients were reported (Arellano-Rodrigo *et al*, 2003; Enting *et al*, 2004), most series were retrospective (Tyson *et al*, 2003; Enting *et al*, 2004), used heterogeneous salvage treatment (Tyson *et al*, 2003), heterogeneous drug dose and schedule (Enting *et al*, 2004) or included patients with systemic recurrence (Fischer *et al*, 2006). Furthermore, the series that presented response and survival data superior to those reported here included more patients with favourable prognostic factors (younger age, better PS) or had many patients who had been treated with chemotherapy alone. Patients with PCNSL who relapse following chemotherapy-only regimens that avoid radiotherapy generally have more chemosensitive disease and better responses to salvage chemotherapy. These patients are candidates to salvage irradiation as well, which may influence overall survival. The use of 1-week-on 1-week-off temozolomide schedule combined with the anti-CD20 monoclonal antibody rituximab achieved a 53% objective response rate and a median survival of 14 months in a retrospective small series that was biased by many of the above-mentioned factors (Enting *et al*, 2004). However, the short median PFS (2.2 months) observed in this series suggests that overall survival was influenced more by treatment administered after temozolomide–rituximab failure than by the study combination itself. Furthermore, the median PFS obtained by temozolomide and rituximab in this study is similar to our experience with conventionally administered temozolomide monotherapy, suggesting that no additional benefit is derived by the addition of rituximab or from a dose intensification of temozolomide. We believe that temozolomide is an excellent candidate agent for further development as a treatment for PCNSL for several reasons: it is well tolerated, even in elderly or poor PS patients; it exhibits additive cytotoxic activity with radiotherapy and in fact may be a radiosensitising agent; and its noncumulative and modest toxicity makes it potentially useful as an agent for induction, consolidation and maintenance therapy.

The present trial represents a simple and effective model for evaluating single agents in this rare disease. This strategy can be employed to identify quickly active new agents that can subsequently be incorporated into therapeutic approaches to the initial management of PCNSL aimed at improving disease control and survival.

## Figures and Tables

**Table 1:** Summary of patient characteristics at baseline

**Patient**	**Age**	**Gender**	**PS**	**Histotype**	**First line**	**Number of lines**	**Lesions**	**RT**	**Failure**	**TFTF**	**OR**	**Duration**	**Survival**
1	66	Male	3	DLC	MVP	2	Single	+	R	5	CR	70.0+	74.0+
2	54	Male	2	DLC	MVP	1	Multiple	+	R	19	PR	2.5	2.5
3	61	Female	0	DLC	MA	1	Multiple	−	R	10	PD		2.0
4	60	Male	2	DLC	MBAVm	1	Multiple	+	R	23	ED		1.5
5	57	Male	3	DLC	MATI	1	Single	−	PD	4	PD		3.5
6	52	Male	3	DLC	CVOD/BVAM	1	Multiple	+	R	11	ED		1.0
7	62	Female	0	DLC	MATI	1	Multiple	+	R	14	PD		1.5
8	54	Male	1	LP	M	1	Multiple	+	PD	10	SD	16.5	54.5+
9	68	Male	2	DLC	none	0	Multiple	+	R	99	PD		4.5
10	64	Male	1	DLC	MATI	1	Single	+	R	12	SD	2.0	2.0
11	51	Male	1	DLC	MA	1	Multiple	+	R	39	CR	2.0	19.0
12	54	Female	2	DLC	F-MACHOPn	2	Multiple	+	R	130	PD		3.5
13	54	Male	2	DLC	MAI	4	Single	+	R	28	PD		2.5
14	64	Female	1	DLC	MVP	1	Multiple	+	R	24	PD		16.0
15	61	Female	2	UN	M	1	Single	+	R	19	CR	17.5+	18.0+
16	62	Male	0	DLC	MVP	1	Single	−	R	14	PD		27.5
17	54	Female	1	DLC	VPAL	1	Multiple	+	R	20	SD	9.5	9.5
18	81	Male	1	UN	MVP	1	Single	−	R	44	CR	6.0	22.5+
19	48	Male	2	UN	MVP	1	Multiple	+	PD	6	ED		1.0
20	66	Male	2	DLC	A	1	Multiple	+	R	48	ED		1.0
21	47	Male	0	DLC	A	1	Single	+	R	14	SD	5.0	25.5+
22	69	Male	4	DLC	MA	1	Single	+	R	38	CR	4.5	7.0
23	56	Female	3	DLC	MA	1	Multiple	+	R	18	PD		0.5
24	75	Male	3	DLC	none	0	Multiple	+	R	21	PR	6.5	9.0
25	59	Female	3	DLC	MATI	1	Multiple	+	PD	9	PD		2.0
26	34	Male	2	DLC	MA,P, PBSCT	3	Multiple	+	PD	20	CR	1.0	5.5
27	36	Male	2	DLC	MABET	2	Multiple	+	PD	1	ED		1.0
28	58	Male	3	DLC	MATI	1	Single	+	R	9	ED		1.5
29	51	Female	2	DLC	M, Me, N, R, PBSCT	1	Multiple	−	R	20	PD		2.0
30	59	Female	2	DLC	MATI	1	Multiple	+	R	8	PD		5.0
31	73	Female	3	DLC	MATI	1	Multiple	+	R	28	CR	20.0+	22.0+
32	54	Female	3	DLC	MATI	1	Multiple	+	R	54	CR	9.5	13.5
33	64	Male	2	DLC	MATI	1	Multple	+	R	12	PD		2.5
34	65	Male	3	HG	MA	2	Multiple	+	PD	8	CR	7.0+	14.0+
35	72	Male	2	DLC	—	—	Single	+	PD	8	SD	5.0	5.5
36	65	Male	2	DLC	M	1	Multiple	+	R	10	PD		1.5

Abbreviations: A=cytarabine; B=carmustine; CR=complete response; D=dexamethasone; DLC=diffuse large B cells; E=etoposide; ED=early death; F=fluorouracil; HG=high grade; I=idarubicin; LP=lymphoplasmacytic; Me=melphalan; M=methotrexate; N=novantrone; O=doxorubicin; OR=overall response; P=procarbazine; PBSCT=peripheral blood stem cell transplantation; PD=progression; Pn=prednisone; PR=partial response; PS=performance status; R=recurrence; Ri=rituximab; RT=radiotherapy; SD=stable disease; T=thiotepa; TFTF=time to first treatment failure; UN=unclassified; V=vincristine.Vm: teniposide; L=lomustine.
